# Upregulation of the APOBEC3 Family Is Associated with a Poor Prognosis and Influences Treatment Response to Raf Inhibitors in Low Grade Glioma

**DOI:** 10.3390/ijms221910390

**Published:** 2021-09-27

**Authors:** Cheng Luo, Songmao Wang, Weijie Liao, Shikuan Zhang, Naihan Xu, Weidong Xie, Yaou Zhang

**Affiliations:** 1 China State Key Laboratory of Chemical Oncogenomics, Tsinghua Shenzhen International Graduate School, Shenzhen 518055, China; luoc19@mails.tsinghua.edu.cn (C.L.); wsm19911020@163.com (S.W.); liaoweijie@sz.tsinghua.edu.cn (W.L.); gavinz1987@aliyun.com (S.Z.); xu.naihan@sz.tsinghua.edu.cn (N.X.); xiewd@sz.tsinghua.edu.cn (W.X.); 2 Department of Biomedical Engineering, Tsinghua University, Beijing 100084, China; 3 Key Lab in Healthy Science and Technology of Shenzhen, Tsinghua Shenzhen International Graduate School, Shenzhen 518055, China; 4 School of Life Sciences, Tsinghua University, Beijing 100084, China; 5 Open FIESTA Center, Tsinghua University, Shenzhen 518055, China

**Keywords:** APOBEC3 family, glioma, prognostic prediction, Ras/MAPK pathway, immune response

## Abstract

Apolipoprotein B mRNA-editing enzyme catalytic polypeptide-like 3 (APOBEC3) has been identified as a group of enzymes that catalyze cytosine deamination in single-stranded (ss) DNA to form uracil, causing somatic mutations in some cancers. We analyzed the APOBEC3 family in 33 TCGA cancer types and the results indicated that APOBEC3s are upregulated in multiple cancers and strongly correlate with prognosis, particularly in low grade glioma (LGG). Then we constructed a prognostic model based on family expression in LGG where the APOBEC3 family signature is an accurate predictive model (AUC of 0.85). Gene mutation, copy number variation (CNV), and a differential gene expression (DEG) analysis were performed in different risk groups, and the weighted gene co-expression network analysis (WGCNA) was employed to clarify the role of various members in LGG; CIBERSORT algorithm was deployed to evaluate the landscape of LGG immune infiltration. We found that upregulation of the APOBEC3 family expression can strengthen Ras/MAPK signaling pathway, promote tumor progression, and ultimately reduce the treatment benefits of Raf inhibitors. Moreover, the APOBEC3 family was shown to enhance the immune response mediated by myeloid cells and interferon gamma, as well as PD-L1 and PD-L2 expression, implying that they have immunotherapy potential. Therefore, the APOBEC3 signature enables an efficient assessment of LGG patient survival outcomes and expansion of clinical benefits by selecting appropriate individualized treatment strategies.

## 1. Introduction

Cancer continues to be a global problem and one of the diseases that poses a serious threat to human health and quality of life. Cancer can be caused by a variety of factors, and treatment differs greatly from person to person. Researchers have recently sought to identify biomarkers that can be used for early diagnosis, combined with patient characteristics, to develop the best treatment regimens and assess prognosis. As high-throughput sequencing technologies advance, this approach will become increasingly important for improving cure rates and quality of life [1]. Early detection and risk assessment of cancer using specific biomarkers are now considered significant methods of cancer remission, which is critical for cancer prevention and treatment.

One important factor in the occurrence of cancer is the genomic instability of normal cells caused by genomic mutations [2]. Usually, the majority of mutations are harmless, while some of them act as “cancer drivers”, which can lead to cell carcinogenesis, such as invasion, metastasis, and the ability to withstand immune surveillance and therapy. It was reported that the expression pattern of the APOBEC3 gene is linked with genetic instability and mutations and the APOBEC3 family was identified as a group of endogenous somatic mutation inducers [3,4]. Seven members of the APOBEC3 family were identified (APOBEC3A-H, excluding E), all of which are encoded by a set of genes on chromosome 22 [5]. The APOBEC3 family of enzymes can deaminate cytosine in single-stranded DNA to produce promutagenic uracil, which is linked to endogenous retroelements, DNA viruses, and RNA viruses [6,7,8]. Moreover, this ‘off-target’ mutation in the genome underpins APOBEC3 family’s association with certain cancers, such as breast [9,10] and bladder cancer [11,12], and it is thought to play a role in cancer development and drug resistance [13]. The majority of research on this family has focused on antiviral activity or retroactive analysis of mutated human genomic sequences; however, its important role in cancers remains unclear.

Central nervous system (CNS) malignant tumor is one of the worst prognosis cancers, and glioma is the most common primary tumor of CNS, accounting for approximately 80% of malignant brain tumors [14,15]. Although diffuse low- and intermediate-grade gliomas collectively constitute low-grade gliomas (LGGs, WHO grades II and III), which are rarer than grade IV (glioblastoma, GBM) [16] due to their highly invasive nature, complete neurosurgical resection is impossible, leading to recurrence and malignant progression, eventually progressing to glioblastoma. Most patients with these gliomas develop glioblastoma and become resistant to chemotherapy [16,17,18], with a 5-year survival rate of approximately 5% [17]. At present, the underlying pathophysiological mechanism of glioma development remains unclear, limiting the efficacy of alternative therapies for glioma. Understanding glioma progression and developing an accurate predictive model will enable more precise diagnosis, early intervention, and effective treatment.

This study report, for the first time, presents the prognostic value and cancer-promoting effect of the APOBEC3 family in LGG. Specifically, we found that the APOBEC3 family promotes cancer and that integrating mRNA levels enables robust risk stratification, which is beneficial for identifying poor glioma subtypes. While high APOBEC3 family expression stimulates Ras/MAPK signal, this results in reduced sensitivity of Raf inhibitors, and the high expression group may benefit significantly from immune checkpoint inhibitors, whereas the low expression group does not. Therefore, APOBEC3, as a new prognostic biomarker, may provide a new strategy for glioma treatment.

## 2. Results

### 2.1. APOBEC3s Are Upregulated in Multiple Cancer Types and Are Associated with Prognosis

**Figure 1:**
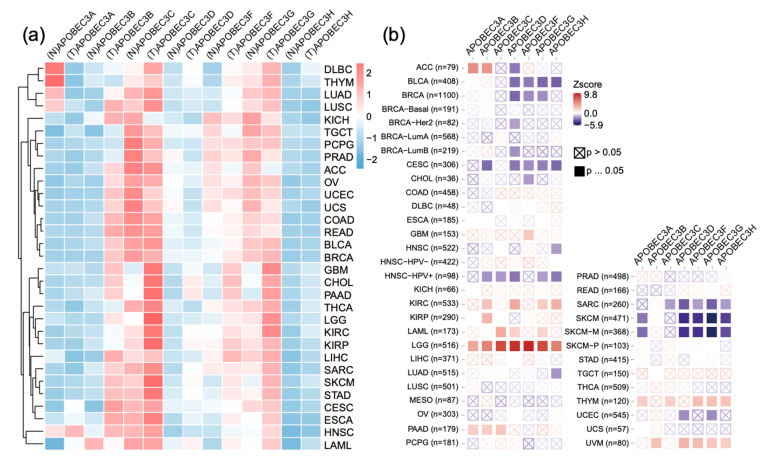
Pan-cancer analysis of the APOBEC3 family expression and survival in TCGA cancers. (**a**) TCGA and GTEx data were integrated to analyze APOBEC3 family expression (TPM) difference between 31 kinds of cancer and normal tissues. (**b**) The relationship between APOBEC3 family expression and survival in 33 TCGA cancers.

In mammalian cells, seven members of the APOBEC3 family have been identified; we combined genotype-tissue expression (GTEx) and The Cancer Genome Atlas (TCGA) to compare the expression of the APOBEC3 family in various cancers (Figure 1a). Our results revealed that all APOBEC3 molecules were upregulated in tumor cells except for APOBEC3A, which was dramatically decreased in DLBC and THYM tumor cells. To identify APOBEC3 family aberrations, we performed mutation and copy number variation analyses in family members. The results indicate that while the mutation of members is uncommon in most cancers, more than 10% of members are mutated in UCEC and SKCM. Furthermore, APOBEC3F and APOBEC3D appear to change in specific cancer environments, such as UCEC (Figure S1a); missense mutation is the main form in all mutation events (Figure S1b). CNV analysis revealed that copy number of the APOBEC3 family varied significantly between cancers, with most samples exhibiting heterozygous amplification in SKCM and LUSC, but more than half exhibiting heterozygous deletion in MESO, OV, and ACC (Figure S1d). APOBEC3 members seem to have the identical copy number alteration pattern, which could be related to their shared locus.

Survival between APOBEC3 family members and different cancers was analyzed using tumor immune estimation resource (TIMER) [19] (Figure 1b). All members were found to be significantly correlated with the prognosis of LGG patients, and high expression of the APOBEC3 family indicated poor prognosis. In contrast, increased expression was associated with good clinical outcomes in metastatic melanoma except for APOBEC3B and APOBEC3C. These results suggest that the APOBEC3 family is heterogeneous in various tumors and is associated with the survival of patients, particularly in low-grade gliomas.

### 2.2. High Expression of the APOBEC3 Family in LGG Is Associated with Malignant Progression

**Figure 2:**
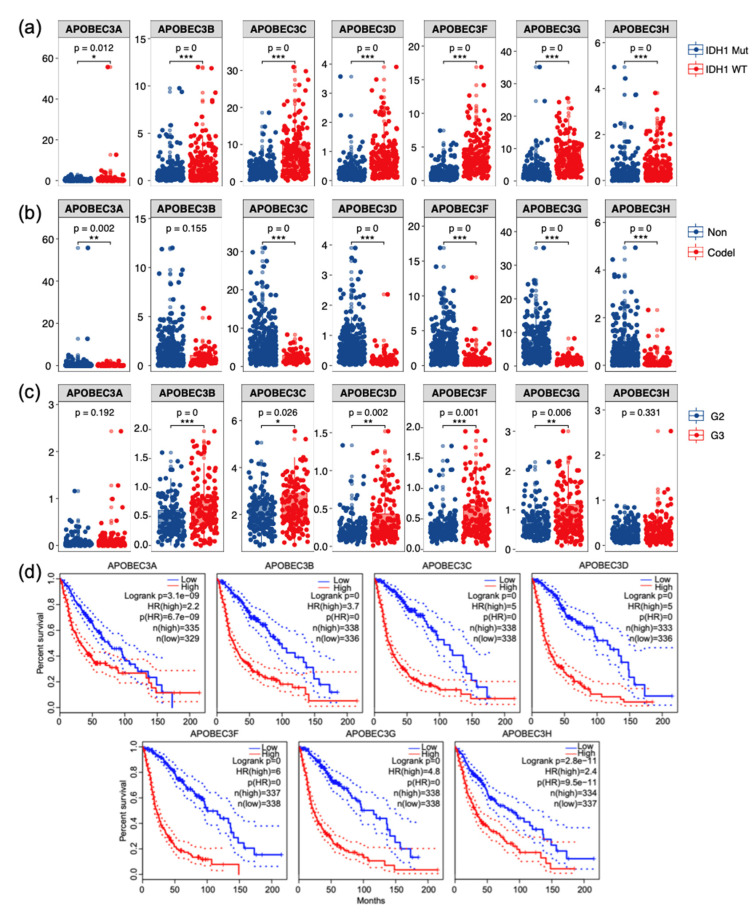
Prognostic features and expression of APOBEC3s in gliomas. (**a**) The expression of APOBEC3 family in the IDH1 wild type group was higher than mutant group. (**b**) The expression of APOBEC3 family in 1p/19q non-codeletion group was significantly higher than codeletion group. (**c**) The expression of APOBEC3 family was upregulated with the increase of tumor grade in LGG. (**d**) The survival curves of APOBEC3 family genes in glioma patients. * *p* < 0.05, ** *p* < 0.01, *** *p* < 0.001.

Some molecular markers were developed for glioma, and some biomarkers are used clinically, such as IDH1 mutation and 1p/19q co-deletion. We found that the expression of member genes was higher in the IDH1 wild type group (Figure 2a) and 1p/19q non-codeletion group (Figure 2b), which indicated poor prognosis. We analyzed the expression of APOBEC3 family members in LGG patients with varying WHO grades and found that, as the tumor grade increased, the expression of all family members increased (Figure 2c). Using the Kaplan–Meier test, we evaluated the association between family members’ expression and prognosis in LGG patients. We found that patients with high expression of APOBEC3 members have a remarkable worse outcome (*p* < 0.0001) (Figure 2d), and similar findings were found in GSE4217 and GSE4412 (Figure S2). These findings demonstrated that the expression of APOBEC3 family in LGG increased with tumor malignancy and was significantly linked to patient prognosis across multiple cohorts.

### 2.3. Construction of the Assessment Model for Prognosis of LGG Survival

**Figure 3:**
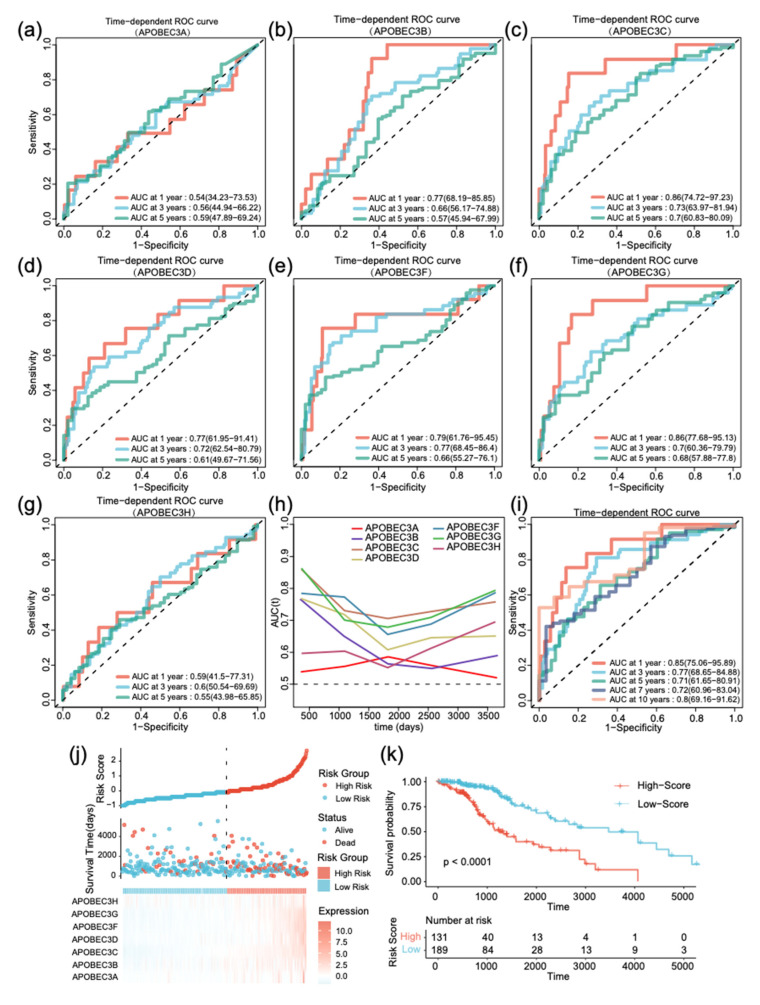
Development of APOBEC3 signature and survival prediction of patients with low-grade gliomas in TCGA training cohort. Time-dependent ROC curve when using (**a**) APOBEC3A, (**b**) APOBEC3B, (**c**) APOBEC3C, (**d**) APOBEC3D, (**e**) APOBEC3F, (**f**) APOBEC3G, and (**g**) APOBEC3H alone to build risk prediction model. (**h**) The area under ROC curve (AUC) of prognosis prediction using different APOBEC3 family members in TCGA data set. (**i**) Time-dependent ROC curve of APOBEC3 signature at 1, 3, 5, 7, and 10 years in TCGA training cohort. (**j**) Distribution of risk score, survival time, and gene expression panel. (**k**) Kaplan–Meier curves of overall survival (OS) in low-grade glioma based on risk score.

Due to the superior performance of the APOBEC3 family in survival curves of prognosis in LGG, we built a multigene panel for predicting the survival of low-grade glioma with family members. The risk score of each patient was estimated based on the expression of these genes and their corresponding coefficients obtained by univariate or multivariate Cox regression analysis: risk score = (1.591*expression of APOBEC3A) + (1.147*expression of APOBEC3B) + (1.582*expression of APOBEC3C) + (1.790*expression of APOBEC3D) + (1.480*expression of APOBEC3F) + (1.299*expression of APOBEC3G) + (0.449*expression of APOBEC3H). Based on ROC analysis results, patients were categorized into significant risk groups based on the optimized risk value. Figure 3a–g revealed the area under the curve of time-dependent ROC when a single gene expression of APOBEC3 family members is used. The results indicate that when APOBEC3C and APOBEC3G are utilized as univariate risk signatures separately, the model has almost the same excellent prediction ability (AUC of APOBEC3C at 1, 3, and 5 years were 0.86, 0.73, and 0.7, respectively; AUC of APOBEC3G at 1, 3, and 5 years were 0.86, 0.7, and 0.68, respectively). Figure 3h shows that the prediction performance of APOBEC3F will be better than other members for the first 1000 days, followed by a sharp decline.

Moreover, we attempted to integrate all family members and found that the model outperformed any gene used alone (AUC at 1, 3, and 5 years were 0.85, 0.77, and 0.71, respectively) (Figure 3i). Figure 3j illustrates the distribution of patient risk scores, survival time, and heatmap of APOBEC3 family profiles in each patient. Kaplan–Meier survival curve indicates that clinical outcome in high-risk group was significantly worse than in low-risk group (*p* < 0.0001) (Figure 3k). In summary, although a single-gene signature based on the APOBEC3 family can partially predict the prognosis of LGG, APOBEC3 signature that includes all members performs better in terms of prognosis prediction.

### 2.4. APOBEC3s Signature Has a Good Performance in Verification Cohort and Clinical Nomogram

**Figure 4:**
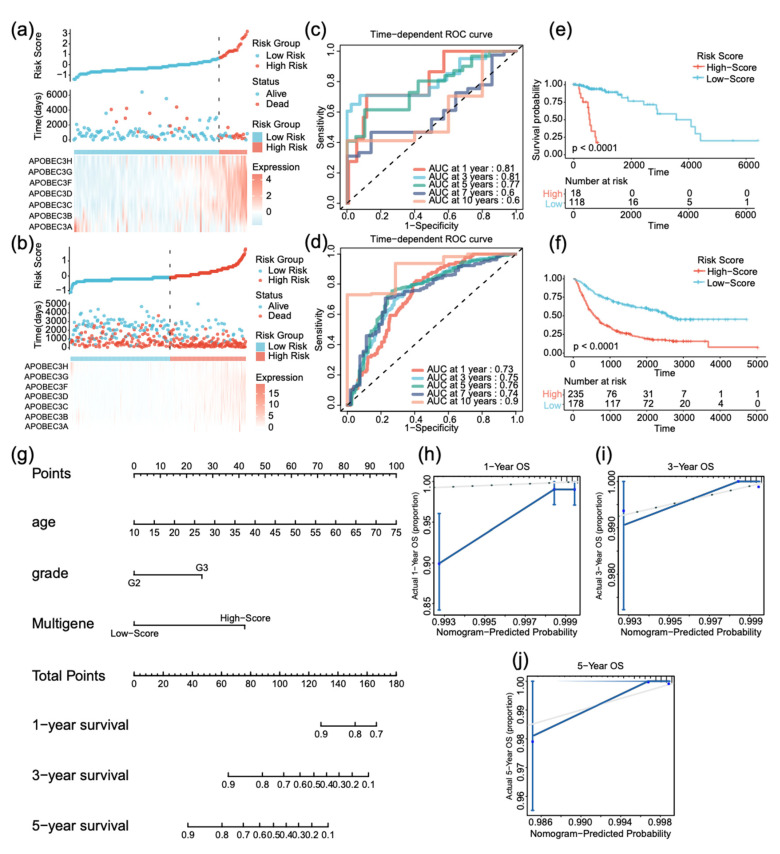
Validation of APOBEC3 signature in TCGA and CGGA cohorts and nomogram construction. (**a**,**b**) Distribution of risk score, survival time, and gene expression panel in TCGA and CGGA validation cohort. (**c**,**d**) ROC curve of risk gene signature at 1, 3, 5, 7, and 10 years in TCGA and CGGA cohort. (**e**,**f**) Kaplan–Meier curves of OS based on risk score in TCGA and CGGA cohort. (**g**) Nomogram predicting 1-, 3-, and 5-year OS for low-grade glioma patients based on APOBEC3 signature and other clinicopathological parameters. (**h**–**j**) The calibration curves of nomogram in predicted and observed 1-, 3-, and 5-year OS.

To validate the performance forecast of multigene signature, we used patient data from TCGA and the Chinese Glioma Genome Atlas (CGGA) as internal and external verification cohorts, respectively. According to the expression of the APOBEC3 family, patients were divided into high-risk (N = 18 in TCGA, N = 235 in CGGA) and low-risk (N = 118 in TCGA, N = 178 in CGGA) groups using the same coefficients (Figure 4a,b). The AUC for predicting prognosis at 1, 3, and 5 years in the verification cohort was 0.81, 0.81, and 0.77, respectively, in TCGA (Figure 4c), whereas it was 0.73, 0.75, 0.76 in CGGA (Figure 4d). In two cohorts (Figure 4e,f, *p* < 0.0001), Kaplan–Meier survival analyses revealed that patients in low-risk group had significantly better OS than those in high-risk group.

The prognostic nomogram is a quantitative method for clinicians to predict the survival of LGG patients. To increase the clinical application value of APOBEC3 signature, we conducted univariate and multivariate Cox regression analyses on various clinical factors. According to *p* < 0.05, we found that APOOBEC3 signature, age, and grade were independent prognostic factors (Table 1). Nomogram was constructed based on these factors to predict 1-year, 3-year, and 5-year survival probability of LGG patients (Figure 4g). The calibration plot closely resembled the ideal diagonal curve at 1, 3, and 5 years (Figure 4h–j), and C-indexes of nomogram were 0.811, indicating its reliable performance.

We divided two cohorts into different subgroups based on their clinical and genetic characteristics and examined the predictive performance of risk model in each subgroup (Figures S3 and S4). In any clinical subgroup, the model has excellent discrimination performance (*p* < 0.05).

### 2.5. The High-Risk Group Had a Higher Tumor Mutation Burden and Copy Number Variation Frequency

We previously speculated that high APOBEC3 family member expression was associated with a poor prognosis in LGG patients (Figure 2), which was confirmed in APOBEC3 signature and revealed that all family members were upregulated in the high-risk group. As a result, high- and low-risk groups we obtained precisely correspond to APOBEC3 family’s high- and low-expression groups. Therefore, due to the biochemical function of the APOBEC3 family itself, we need to examine the molecular genetic changes in two different expression groups of low-grade gliomas.

APOBEC3 family has been implicated in promoting mutation in other cancers. As a result, we used gene mutation and copy number analysis to elucidate the influence of APOBEC3 family on genetic level of LGG. The mutation rates of IDH1, TP53, and ATRX were higher than 40% in both groups (Figure S5a,b). It is worth noting that 10% of NF1 mutations were found in the high expression group, but disappeared in the low group, and the NF1 gene mutation leads to Ras/MAPK pathway dysfunction [20]. Following that, we discussed differences in tumor mutation burden (TMB) and mutation times between the two expression groups, with the high expression group having a higher TMB (*p* = 0.014) (Figure S5c–e).

GISTIC2.0 algorithm was used to evaluate CNV of the two groups, and it was discovered that high-risk group experienced more severe and frequent changes. This group contained three copy number amplification regions: 1q32.1, 7p11.2, and 12q14.1 (Figure S5f–i). MAPKAPK2, ATP2B4, IL10, and IL24 are all found on 1q32.1, involved in the p38 MAPK pathway and inflammation [21,22]. The epidermal growth factor receptor-related proteins (EGFR) are located on 7p11.2, while AGAP2, CDK4, and USP15 are found on 12q14.1. All of these findings suggest that APOBEC3 overexpression in LGG causes increased mutation and copy number amplification in specific regions, as well as changes in the expression of related genes, especially Ras/MAPK signaling genes.

### 2.6. DEGs between Two Different APOBEC3s Expression Groups Are Associated with Immune Response Pathway

**Figure 5:**
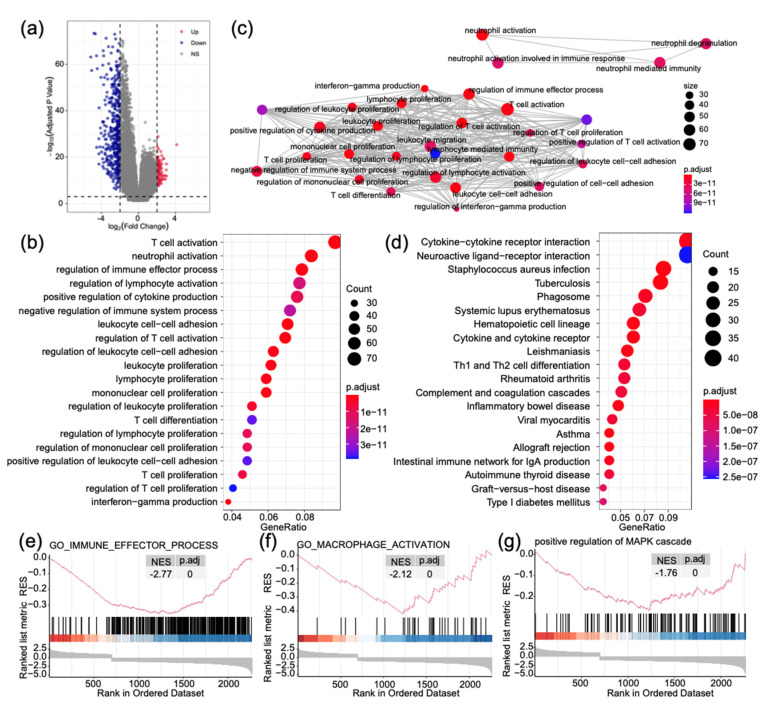
Gene function enrichment analysis identified biological pathways and processes associated with a high expression patient. (**a**) Volcano plot of differentially expressed genes between high- and low-expression patients. (**b**) Dot plot of gene ontology (GO) enriched terms colored by *p*-values. (**c**) Significant GO items were clustered according to biological function. (**d**) Dot plot of Kyoto Encyclopedia of Genes and Genomes (KEGG) enriched terms colored by *p*-values. (**e**–**g**) Gene set enrichment analysis between high- and low-expression patients. RES, running enrichment score. NES, normalized enrichment score.

Using DESeq2 algorithm, we analyzed differentially expressed genes between high- and low-expression groups from TCGA cohort, identifying 1123 upregulated and 2292 downregulated genes (logFC > 1, *p* < 0.001). The log2 of enrichment ratio and −log10 of adjusted *p*-value were visualized in the volcano plot (Figure 5a).

Gene Ontology (GO) analysis indicated that these genes could be categorized into lymphocyte activation and immune response, including T cell activation, neutrophil, and lymphocyte activation (Figure 5b). As displayed in Figure 5c, the items with high significance are clustered in the immune response is related to interferon gamma, myeloid-derived cells, and cytokine. Kyoto Encyclopedia of Genes and Genomes (KEGG) analysis revealed that DEGs were mainly linked to essential biological processes, including cytokine–cytokine receptor interaction, neuroactive ligand–receptor interaction, Th1 and Th2 cell differentiation, and complement and coagulation cascades (Figure 5d).

Fold changes of mRNA expression levels of DEGs between high-risk and low-risk groups were calculated and pre-ranked. Gene Set Enrichment Analysis (GSEA) analysis unveiled that altered genes were significantly enriched in neuron and nervous system development. We also found that the low expression group was significantly associated with immune effector process (NES = −2.77, *p*.adj < 0.0001, Figure 5e), macrophage activation (NES = −2.12, *p*.adj < 0.0001, Figure 5f), and positive regulation of MAPK cascade (NES = −1.76, *p*.adj < 0.0001, Figure 5g).

The high-frequency change of immune response-related signaling pathways in enrichment results suggests a huge difference in immune landscapes between solid tumors of two different APOBEC3 groups. We selected genes related to chemokines (KEGG_CHEMOKINE_SIGNALING_PATHWAY and WP_CHEMOKINE_ SIGNALING_PATHWAY, total of 354 genes), inflammatory factors (HALLMARK _INFLAMMATORT_RESPONSE, total of 200 genes), and immune activation (GO_BP_ACTIVATION_OF_IMMUNE_RESPONSE, total of 563 genes) through MSigDB database to further analyze APOBEC3 signature and changes of these signaling pathways between two different expression solid tumors.

According to the correlation coefficient, we selected 68 genes (correlation coefficient with any APOBEC3s is greater than 0.6) and APOBEC3 family members to draw a cluster diagram (Figure S6). On the one hand, we found that these genes can be clustered into eight clusters, among which APOBEC3A and APOBEC3B were isolated separately, demonstrating that different members may have different biological functions in low-grade gliomas. On the other hand, we found that these immune response markers include three families: (1) complement, (2) major histocompatibility complex, and (3) C-X-C motif chemokine ligand and receptor. All of these immune families may be closely related to APOBEC3 family in LGG, indirectly regulating the immune response of patients and the immune landscape of solid tumors.

### 2.7. APOBEC3s Members Affect Myeloid Cells and Interferon Gamma Activation in LGG

**Figure 6:**
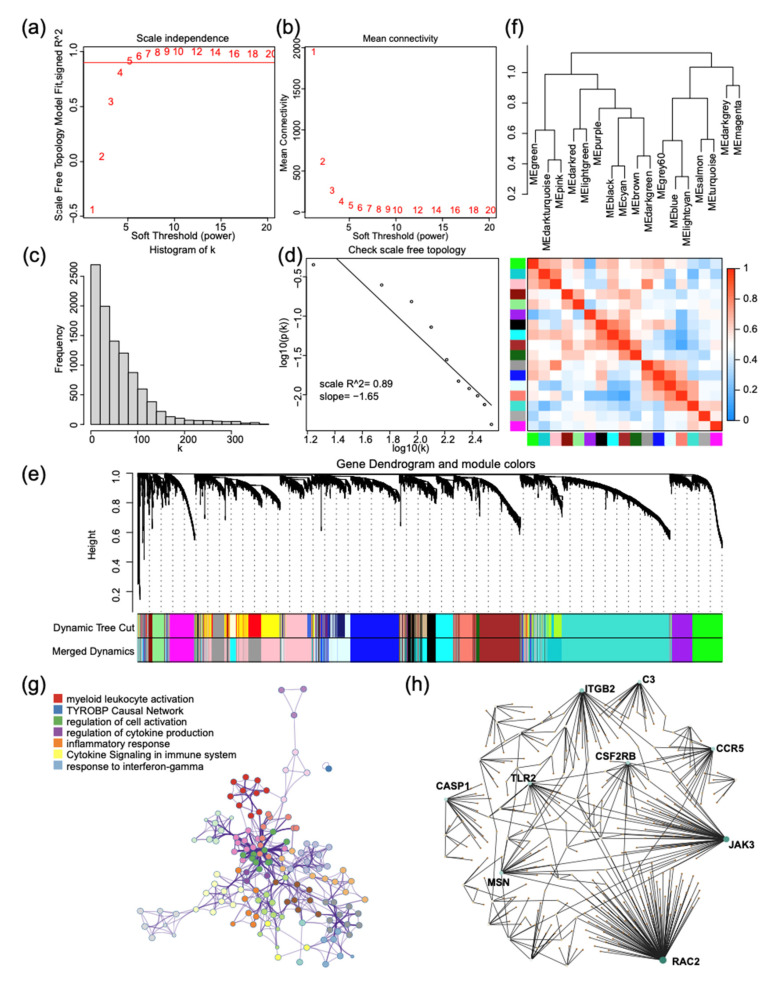
Construction of weighted risk stratification gene co-expression network and functional enrichment analysis. (**a**,**b**) The scale-free fit index for soft thresholding powers. (**c**,**d**) Selecting the soft threshold of 5, checking the node connection number, and verifying the network connectivity of a scale-free network. (**e**) Combining the modules with small dissimilarity, 17 weighted gene co-expression sub-networks were obtained by a dynamic tree cut algorithm. (**f**) The correlation between network modules. (**g**) Gene ontology analysis of all module genes within five members colored by specific cluster ID. (**h**) PPI network was constructed by screening the hub genes according to the fold change of DEGs in brown mod.

As current results suggest, we need to study the role of each family member in LGG individually rather than as a whole. We selected 20,000 genes based on their median absolute deviation and transformed 520 LGG expression profiles (TCGA) into gene co-expression networks using WGCNA package, as described previously. We selected a soft threshold (beta = 5) to build a scale-free network and check the mean connectivity of the network (Figure 6a,b). Figure 6c,d is used to verify the network node connection statistics and scale-free distribution. The fractional-step algorithm is used to construct modules and calculate the correlation between them (Figure 6e), and finally, 17 different co-expression modules are obtained (Figure 6f).

Through enrichment analysis of WGCNA co-expression modules, we found that the APOBEC3 family was located in three different co-expression modules. APOBEC3C, APOBEC3D, APOBEC3F, APOBEC3G, and APOBEC3H are located in the brown module, and the remaining two are located in separate two modules, consistent with our conclusion of correlation coefficient clustering above. In other words, APOBEC3A and APOBEC3B may play different roles in LGG than other members. Following that, we performed a functional enrichment analysis of brown module genes, and found that they were mainly involved in body’s immune response, including myeloid leukocyte activation, cytokine production, and response to interferon gamma (Figure 6g). We defined the brown module’s hub gene as regulated by the APOBEC3 family, which has a logFC of more than 1.5. The protein–protein interaction network of key genes was mapped, and the most cohesive node was found (Figure 6h). We discovered that RAC2 and JAK3 are responsible for two largest subnetworks. Immune cells and their effector molecules use JAK3 to transmit signals from cell surface to nucleus. As a Ras kinase, RAC2 regulates the classic p21-Raf-MEK-ERK pathway and cooperatively activates interferon gamma production [23,24]. TLR2 was also demonstrated to promote interferon gamma production and then mediate T cell activation [25].

Furthermore, we performed enrichment analysis on all hub genes. We discovered that four of the top five pathways are involved in the MHC II protein complex and its receptor activity, which is an immune family that we found in the previous section (Table 2). These findings suggest that the APOBEC3-regulated molecular network may influence glioma cell progression via the Ras/MAPK signaling pathway and regulate interferon gamma production, macrophage activation, and immune response.

### 2.8. High APOBEC3 Levels Associated with Macrophage Infiltration and Upregulation of Immune Checkpoint

**Figure 7:**
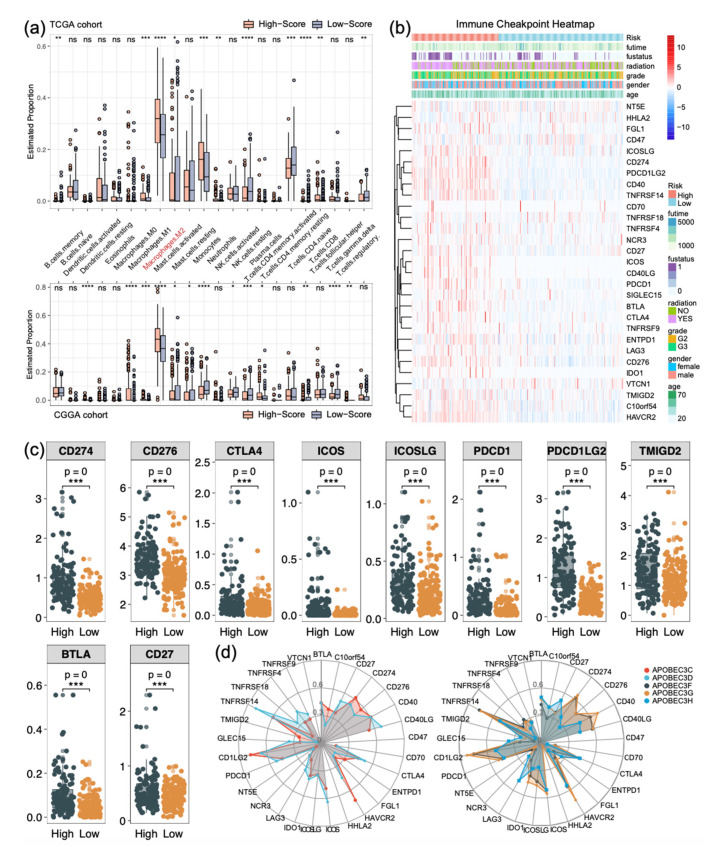
The relationship between different APOBEC3 expression groups and immune profiles in low-grade glioma. (**a**) Boxplot of the CIBERSORT algorithm for evaluating the proportion of 22 kinds of immune cell infiltrations in high- and low-expression solid tumors. (**b**) The heatmap integrated the clinical features and immune checkpoint expression in the TCGA dataset. (**c**) Most immune checkpoints were activated in the high expression group in TCGA glioma patients. (**d**) Radar graph of the APOBEC3 family and immune checkpoint based on correlation coefficient. *p* < 0.05, * *p* < 0.01, ** *p* < 0.001, *** *p* < 0.0001, **** *p* < 0.00001.

Based on the above results, we applied the CIBERSORT algorithm to predict the content of immune cells in tumor microenvironment. We evaluated the accuracy of results using a 100-fold permutation test. Patients with *p*-value < 0.05 were selected. The results of TCGA and CGGA are displayed in Figure 7a; patients in the high expression group had a significantly different proportion of M2 types of macrophages both of two cohorts. We analyzed the relationship between the APOBEC3 family expression and proportion of macrophages, monocytes, and NK cell infiltration in the solid tumor using the TIMER 2.0 database (Figure S7). The results showed that APOBEC3C, APOBEC3D, APOBEC3F, APOBEC3G, and APOBEC3H expression were significantly positively correlated with macrophage and monocyte infiltration, particularly APOBEC3C, APOBEC3G, and APOBEC3H, where Rho was even greater than 0.5 (*p* < 0.0001).

Following that, we extended our analysis to 28 immune checkpoint molecules, including B7-CD28 family [26,27], TNF superfamily [28], and others [29,30,31]. TCGA and CGGA results are illustrated in Figure 7b,c and Figure S8a,b, respectively, and Figure 7d displays the radar plot of correlation coefficients between the APOBEC3 family and immune checkpoints. We were surprised that nearly all immune checkpoints revealed great differences among patients with different expression groups (*p* < 0.0001, except for TNFRSF18, VTCN1, and SIGLEC15). These results indicate that immune checkpoint molecules are generally stimulated in APOBEC3 solid tumors with high expression levels, implying that immune checkpoint inhibitors may achieve different clinical benefits for patients with different APOBEC3 family expression levels.

### 2.9. APOBEC3s Affect the Response of Tumor Cells to a Series of Drugs

**Figure 8:**
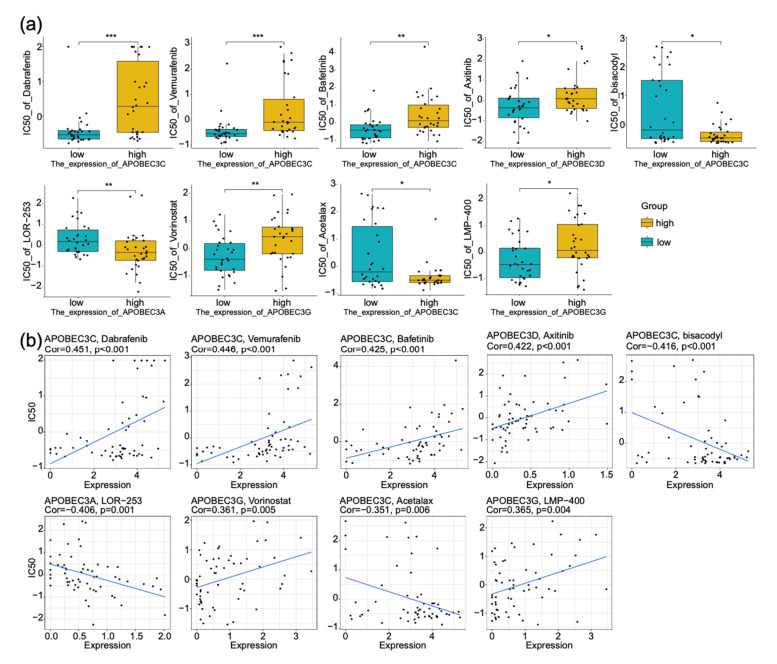
Effect of APOBEC3 family on drug sensitivity of tumor cells based on NCI-60 cell line data. Boxplot (**a**) and scatter plot (**b**) were used to study the relationship between the expression level of APOBEC3 family members and the drug sensitivity of 263 compounds (75 clinically tested and 188 FDA approved drugs) in tumor cells, nine compounds with high correlation were selected to display. *p* < 0.05, * *p* < 0.01, ** *p* < 0.001, *** *p* < 0.0001.

The previous part of this study confirmed the prognostic value of the APOBEC3 family in patients with low-grade glioma. We hope further to elucidate the therapeutic value of APOBEC3 family in cancer. For this reason, we collected drug sensitivity analysis data of all compounds on the NCI-60 cell line panel and RNA sequencing data of cell lines from the National Cancer Institute (NCI) database. We selected 75 clinical trials and 188 FDA-approved drugs to study the relationship between the expression level of APOBEC3 family and IC50 of the above drugs and visualized the most significant nine results (Figure 8a). The Pearson correlation between each gene expression and different drugs was calculated (Figure 8b). The results elaborated that the abundance of the APOBEC3 family was related to the sensitivity of tumor cells to dabrafenib (Cor = 0.451, *p* < 0.001), vemurafenib (Cor = 0.446, *p* < 0.001), bafetinib (Cor = 0.425, *p* < 0.001), axitinib (Cor = 0.422, *p* < 0.001), bisacodyl (Cor = −0.416, *p* < 0.001), LOR-253 (Cor = −0.406, *p* = 0.001), vorinostat (Cor = 0.361, *p* = 0.005), acetalax (Cor = −0.351, *p* = 0.006), and LMP-400 (Cor = 0.365, *p* = 0.004). We were surprised to identify that two of the most significant compounds, dabrafenib, and vemurafenib, are Raf inhibitors. On the one hand, patients with high APOBEC3 family expression may be resistant to the Raf inhibitor therapy. On the other hand, this study supports our hypothesis that a link between the APOBEC3 family and the Ras-Raf-MAPK pathway exists in cancers other than glioma, which has not been reported.

## 3. Discussion

The APOBEC3 family was reported as an endogenous source of somatic mutations. Despite the fact that dysregulation has been linked to cancer, the family was shown to play an important physiological role in protecting cells from endogenous and exogenous DNA-based genotoxins. APOBEC3B deletion was shown to affect neoantigen loads and immune cell compositions in BRCA patients [32]. The APOBEC3 mutational signature is also more prevalent in NSCLC patients who have experienced long-term clinical benefits following immunotherapy [33]. In this study, we elucidated the role of the APOBEC3 family in low grade glioma that can predict prognosis and identified patients who may not benefit from immunotherapy.

Previous studies revealed that APOBEC3 fuels subclonal diversification and cancer heterogeneity in some cancers [34]. Upregulation of APOBEC3s (particularly the B) in breast cancer leads to an increase in TP53 and PIK3CA mutations [18,35], which is linked to tumorigenesis, poor prognosis, and resistance to many anti-cancer drugs [36]. Similar findings were found in NSCLC, which is associated with a poor prognosis [37]. Besides, increased APOBEC3G expression makes cells more sensitive to cisplatin, which may contribute to the improved patient outcomes seen in HNSCC [38]. Some of these findings are consistent with our results, which found that increased APOBEC3 family expression is linked to an increased risk of LGG patients and reduced tumor cell response to a number of drugs, including dabrafenib and vemurafenib. In addition, TP53 and NF1 mutations were more common in high expression samples than low samples (55% vs. 46% and 10% vs. 1% respectively), but there was no discernible difference in PIK3CA expression. TP53 mutation is linked with more aggressive disease and poorer patient outcomes in many cancers [39,40], particularly in LGG [41]. Moreover, somatic NF1 mutations may be critical drivers in multiple cancers [42]. Therefore, high-APOBEC3 group expression with a high TP53 and NF1 mutations have a worse outcome than groups with low APOBEC3 expression, which is in agreement with our survival results. Our findings on the APOBEC3 family appear to be consistent with previous reports, albeit with some differences in their functions in LGG, necessitating the inclusion of some previously overlooked members.

Outstandingly, we found that the APOBEC3 family upregulates the Ras/MAPK signaling pathway in LGG, which has not been reported before: (1) GSEA indicated that MAPK kinase positive regulation was significantly downregulated in the low expression group (NES = −1.76, *p* < 0.001). (2) In the high expression group, copy number amplification occurred in specific regions encoding numerous Ras/MAPK pathway regulators, including MAPKAPK2, ATP2B4, and USP15. (3) WGCNA clustering was used to obtain the co-expression network regulated by the APOBEC3 family. The PPI network was constructed in such a way that a sub-network with RAC2 at its core was discovered, and RAC2 was identified as a Ras kinase, and was located upstream of the Ras/MAPK pathway. (4) Drug sensitivity analysis of NCI-60 revealed that highly expressed APOBEC3 family tumor cells were resistant to Raf inhibitors.

In addition, we analyzed GO, KEGG, and GSEA of the APOBEC3 family in high- and low-expression groups. The results indicate that the biological function annotation of differential genes involve myeloid-derived cells, secretion of immune-related cytokines, and immune response mediated by interferon gamma. We speculated that these might cause macrophage activation. GSEA confirmed our conjecture that NES of the macrophage positive regulatory pathway was −1.76 (*p* < 0.001) in the low expression group. By analyzing APOBEC3 co-expression network members obtained by WGCNA, we found that the most significant term was the activity of the MHC II protein complex and its receptor, which were mainly involved in presenting foreign peptides [43]. Furthermore, we evaluated the immune infiltration landscapes of various expression groups using the CIBERSORT algorithm. As predicted, M2 macrophages were significantly activated in the high expression group, and most immune checkpoints were upregulated. All of these results suggest that APOBEC3 upregulation in low grade glioma can stimulate macrophages through interferon gamma and activation of myeloid-derived cells, leading to tumor immune response and accelerating tumor cell immune escape by upregulating immune checkpoints. Finally, the relationship between the APOBEC3 family gene expression and macrophage and monocyte infiltration ratio was analyzed by the TIMER 2.0 database. The results revealed a high correlation between the APOBEC3 family gene expression and the macrophage and monocyte infiltration ratio, confirming our conclusion.

Immunotherapy has been shown to be a viable treatment option for advanced or aggressive cancers [44]. Given that the overall survival of LGG patients treated with immunotherapy is still very low [45], identifying patients who will benefit the most from these treatments is critical, but we have yet to develop a reliable model for predicting immunotherapy response and overall survival. Our work looked at the relationship between APOBEC3 expression and immunotherapy biomarkers, such as PD-L1, CTLA-4, and LAG-3, and found that the high expression group had significantly higher levels than the low expression group, implying a better response [46]. Recently, the FDA approved TMB high (TMB-H or TMB ≥ 10) as a biomarker for immune checkpoint inhibitor treatment of solid tumors. We also found differences in TMB between the two expression groups, with the high expression group having a higher TMB (*p* < 0.05), which indicated that TMB could help explain why APOBEC3s affects immunotherapy prognosis at some point.

It was reported that some biomarkers can predict the prognosis of LGG patients, such as the TAM signature and STEAP family. TAM signature is a creative model to predict the prognosis of glioma based on scRNASeq, which eliminate the deviation of the bias of bulk RNAseq data due to the mixed cell types in a tumor [47]. STEAP members have been identified as important metalloreductases in vivo and been shown to play a role in iron homeostasis [48]. They have also been shown to have precise performance in predicting the outcomes of glioma patients [49]. However, the STEAP family focused on survival time rather than response to immunotherapy. In our study, the predictive value of APOBEC3s was comparable with the STEAP family, and it may be a better predictor of OS than the TAM signature, the high APOBEC3 expression groups are more likely to benefit from immunotherapy. These findings could aid clinicians in identifying patients who are most likely to benefit from immunotherapy and, in the future, developing personalized immunotherapy regimens for glioma patients. However, due to the study’s limitations, we should confirm the APOBEC3 signature using a more independent glioma cohort. Additionally, we need to confirm APOBEC3 family’s role in glioma via in vivo and in vitro experiments. Our findings may provide some clues for future research, focusing on the cancer promoting function of APOBEC3s in glioma.

## 4. Materials and Methods

### 4.1. Publicly Available mRNA Data and Immune Gene Sets

Our study incorporated data from two publicly available datasets. TCGA RNA-seq data (FPKM value) of samples from patients with LGG (Illumina HiSeq 2000) were acquired from Genomic Data Commons (GDC) (http://portal.gdc.cancer.gov. accessed on 23 April 2021). According to the whole survival time, age, radiotherapy status, and glioma grades, 420 patients were collected and randomly (in a 7:3 ratio) categorized as training and internal validation sets. A total of 529 glioma data were downloaded with complete clinical data and molecular subtyping data (IDH1 mutation, 1p19q codeletion, and MGMT methylation) from the Chinese Glioma Genome Atlas (CGGA) (http://www.cgga.org.cn. accessed on 26 April 2021) to serve as external validation sets.

### 4.2. Construction and Verification of APOBEC3 Signature

According to mRNA expression of APOBEC3 genes, a stepwise Cox proportional hazards regression model was used. Risk score formula was calculated by considering the expression of optimized genes and the estimated Cox regression coefficients: risk score = (exp Gene1 × coef Gene1) + (exp Gene2 × coef Gene2) + … +(exp Gene7 × coef Gene7). Patients with LGG were classified into a high-risk or low-risk group by ranking the given risk score. R package “timeROC” was used to test the time-dependent receiver operating characteristic curve (ROC) [50]. The difference of overall survival (OS) between the two groups in the three cohorts was assessed using the Kaplan–Meier method and the two-tailed log-rank test. A Cox proportional hazards regression model was employed to identify independent prognostic factors. *p* < 0.05 was considered statistically significant.

### 4.3. Construction and Validation of Multigene Containing Nomogram

A nomogram was used to predict the survival probability by specific clinical parameters [51]. We constructed a nomogram containing multigene signatures and other independent prognostic factors. The nomogram was calibrated at 1-year, 3-year, and 5-year intervals using R package “rms”. The decision curve analysis (DCA) was used to assess the clinical application benefits of the multigene panel in TCGA set [52].

### 4.4. Biological Process and Pathway Enrichment Analysis

Using R package “DESeq2” [53], DEGs between high-risk and low-risk groups were identified. Then, different pathways and items were identified between the two risk groups using Gene Ontology (GO) and the Kyoto Encyclopedia of Genes and Genomes (KEGG) enrichment analysis. In addition, we used GSEA [54] to calculate dynamical scores for different enrichment items of high-risk and low-risk groups.

### 4.5. Weighted Gene Co-Expression Network Analysis

R package “WGCNA” [55] was used to perform weighted gene co-expression network analysis (WGCNA) using TCGA LGG expression matrix (FPKM). To build a scale-free network and calculate network topology matrix, a gene expression matrix was weighted by a soft threshold. We used a dimension reduction algorithm to visualize the network module composed of co-expressed genes in glioma samples after clustering with a dynamic cut tree algorithm and merging similar modules.

### 4.6. Evaluation of Immune Microenvironment with CIBERSORT

LM22 signature matrix, which is included in CIBERSORT, was used to estimate the distribution of 22 immune cell types [56]. We ran 1000 iterations in R studio using the script provided in this paper to assess the difference in 22 immune cell infiltrations between high- and low-expression APOBEC3 groups and displayed the results in a landscape map.

### 4.7. Analysis of Gene Mutation and Copy Number Variation

The copy number variation data in the TCGA database were downloaded through “TCGAbiolinks” R package [57], and the risk score and CNV were integrated. SNP6 grch38 annotation file was downloaded in TCGA and analyzed with GISTIC2.0 [58]. Gene mutation data were also obtained from the TCGA database. The occurrence of mutation events was calculated and matched with the risk score. Finally, “maftools” R package was used for visualization [59]. Pan-cancer analysis was conducted through the GSCA database [60].

## 5. Conclusions

In conclusion, the present study demonstrated that the identified APOBEC3 signature may be a reliable prognostic and predictive marker in patients with LGG. Upregulation of the APOBEC3 family expression reduces the treatment benefits of Raf inhibitors and enhances the immune response mediated by myeloid cells and interferon gamma, as well as PD-L1 and PD-L2 expression. These additional and easily usable tests might facilitate personalized treatment and guide clinical decisions.

## Figures and Tables

**Table 1:** Univariate and multivariate Cox analysis identified independent prognostic factor.

Variables	Unicox			Multicox		
	HR	95%CI of HR	*p*	HR	95%CI of HR	*p*
age	1.05	1.03–1.07	0.00	1.05	1.03–1.07	0.000
gender	1.03	0.68–1.57	0.89			
grade	3.09	1.94–4.91	0.00	2.02	1.22–3.34	0.006
multigene	0.27	0.17–0.42	0.00	0.28	0.18–0.45	0.000
radiation	3.08	1.71–5.55	0.00	1.61	0.85–3.06	0.145

**Table 2:** Hub gene enrichment analysis by STRING database.

#Term ID	Description	Strength	FDR
GO:0042613	MHC class II protein complex	2	1.36 × 10^−17^
KW-0491	MHC II	1.96	9.16 × 10^−17^
CL:18630	MHC class II protein complex	2.03	7.31 × 10^−14^
GO:0032395	MHC class II receptor activity	1.93	9.22 × 10^−10^
CL:18633	Peptide antigen assembly with MHC class II protein complex	2.03	5.69 × 10^−7^
CL:10096	Classical antibody-mediated complement activation	1.95	9.43 × 10^−7^
GO:0023026	MHC class II protein complex binding	1.93	3.85 × 10^−5^

## Data Availability

The authors confirm that the data supporting the findings of this study are available within the article and its Supplementary Materials.

## Supplementary Materials

The following are available online at https://www.mdpi.com/article/10.3390/ijms221910390/s1.
