# Supplementary material for: Upregulation of the APOBEC3 Family Is Associated with a Poor Prognosis and Influences Treatment Response to Raf Inhibitors in Low Grade Glioma
Source: Int J Mol Sci. 2021 Sep 27;22(19):10390. doi: 10.3390/ijms221910390 (PMC8508917; doi:10.3390/ijms221910390)
Supplement: Supplementary file 1 [file ijms-22-10390-s001.zip › Supplementary materials S1.pdf]

Supplementary Figure S1

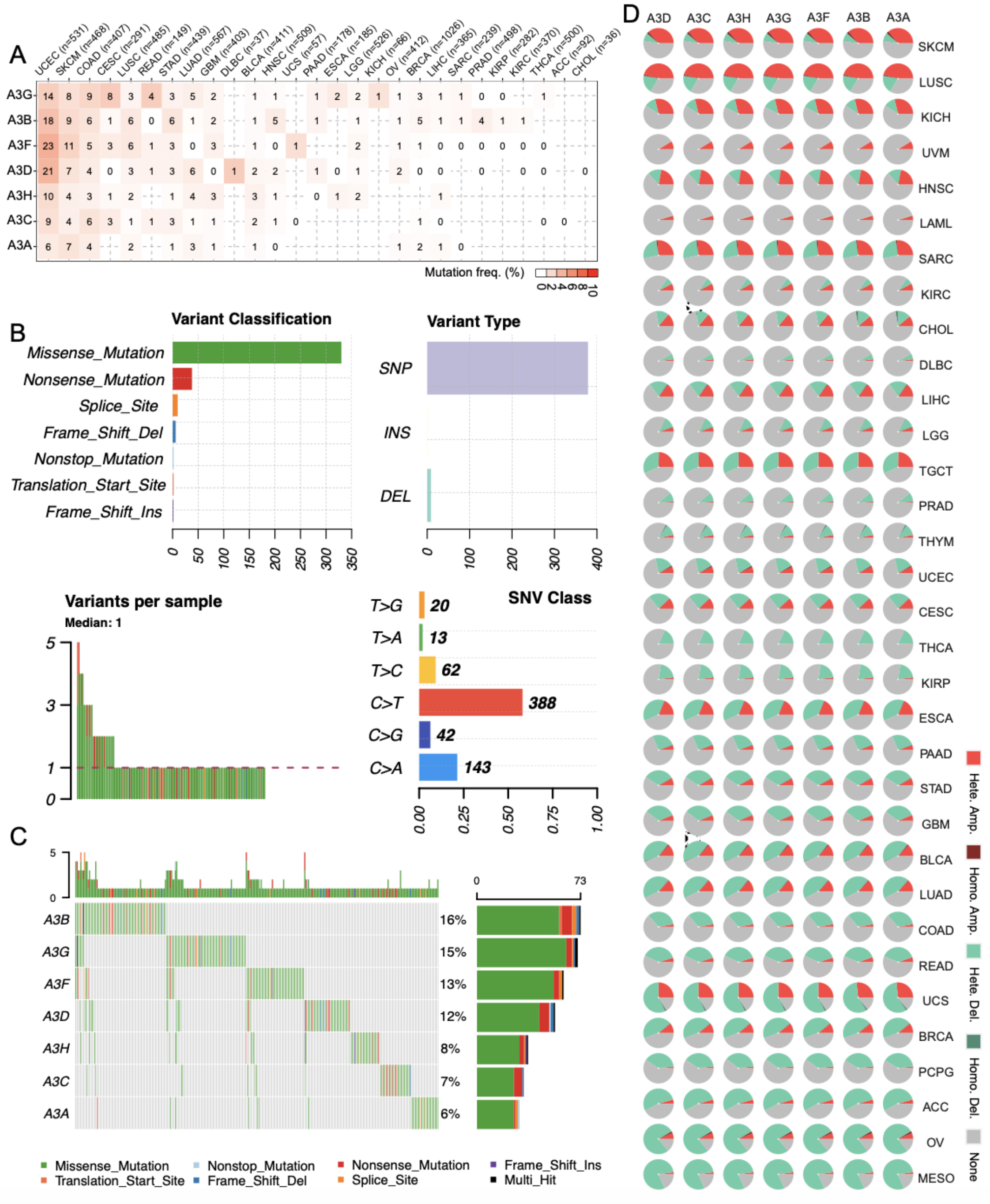

Figure S1: Pan-cancer analysis of APOBEC3 family somatic mutations and copy number variation (CNV) in TCGA cancer. (A) The profile of SNV of APOBEC3 family in cancers. (B) Summary of APOBEC3 family mutation classes in cancer. (C) Oncoplot provides the situation of the SNV of APOBEC3 family in TCGA cancer. (D)Types and proportion of copy number variation in APOBEC3 family in TCGA cancer.
