# Supplementary material for: Upregulation of the APOBEC3 Family Is Associated with a Poor Prognosis and Influences Treatment Response to Raf Inhibitors in Low Grade Glioma
Source: Int J Mol Sci. 2021 Sep 27;22(19):10390. doi: 10.3390/ijms221910390 (PMC8508917; doi:10.3390/ijms221910390)
Supplement: Supplementary file 1 [file ijms-22-10390-s001.zip › Supplementary materials S2.pdf]

Supplementary Figures S2

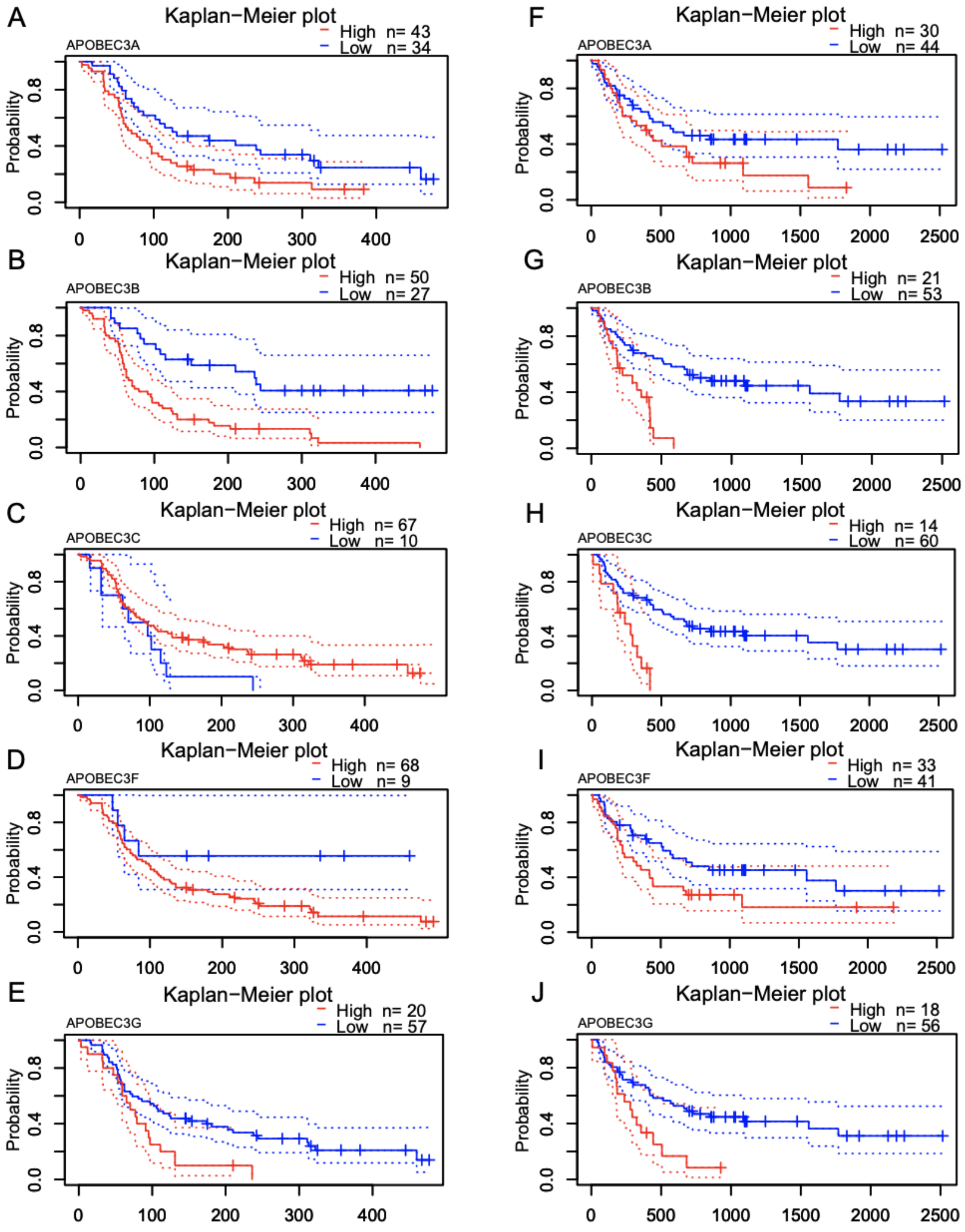

Figure S2: Relationship between APOBEC3 family mRNA level and prognosis of patients in GEO dataset. The survival curve of patients with APOBEC3A (A), APOBEC3B (B), APOBEC3C (C), APOBEC3F (D) and APOBEC3G (E) mRNA expression in GSE4217

astrocytoma data set (n = 77). The survival curve of patients with APOBEC3A (F), APOBEC3B (G), APOBEC3C (H), APOBEC3F (I) and APOBEC3G (J) mRNA expression in GSE4412 glioma data set (n = 74).
