# Supplementary material for: Upregulation of the APOBEC3 Family Is Associated with a Poor Prognosis and Influences Treatment Response to Raf Inhibitors in Low Grade Glioma
Source: Int J Mol Sci. 2021 Sep 27;22(19):10390. doi: 10.3390/ijms221910390 (PMC8508917; doi:10.3390/ijms221910390)
Supplement: Supplementary file 1 [file ijms-22-10390-s001.zip › Supplementary materials S3.pdf]

Supplementary Figures S3

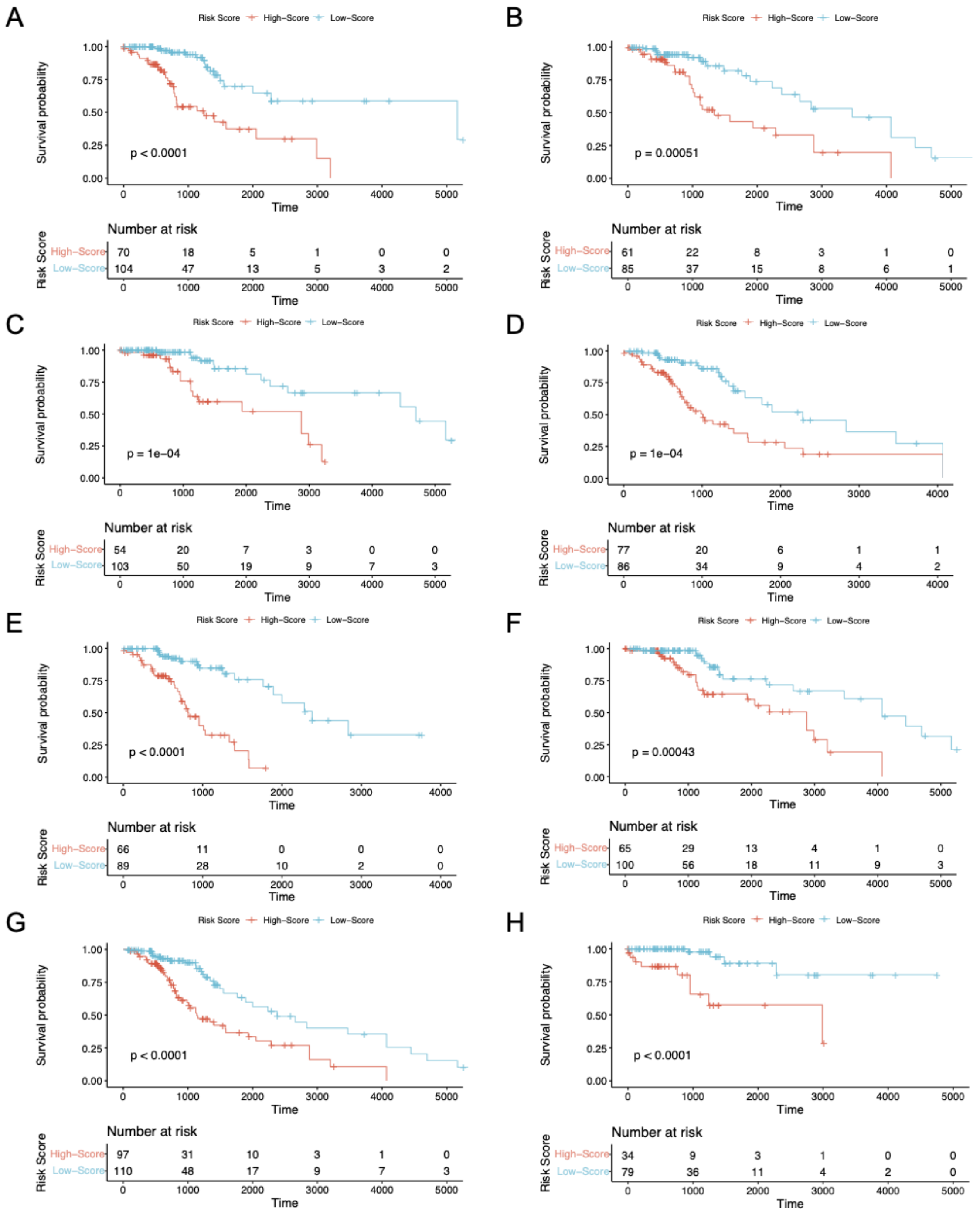

Figure S3: Survival analysis of cases in TCGA training cohort stratified by gender, WHO grades and Radiotherapy. Kaplan-Meier curves of OS in male (A), female (B), WHO II (C), WHO III (D), old (E), young (F), with (G) or without (H) radiotherapy patients based on risk score in low grade glioma population.
