# Supplementary material for: Upregulation of the APOBEC3 Family Is Associated with a Poor Prognosis and Influences Treatment Response to Raf Inhibitors in Low Grade Glioma
Source: Int J Mol Sci. 2021 Sep 27;22(19):10390. doi: 10.3390/ijms221910390 (PMC8508917; doi:10.3390/ijms221910390)
Supplement: Supplementary file 1 [file ijms-22-10390-s001.zip › Supplementary materials S4.pdf]

Supplementary Figures S4

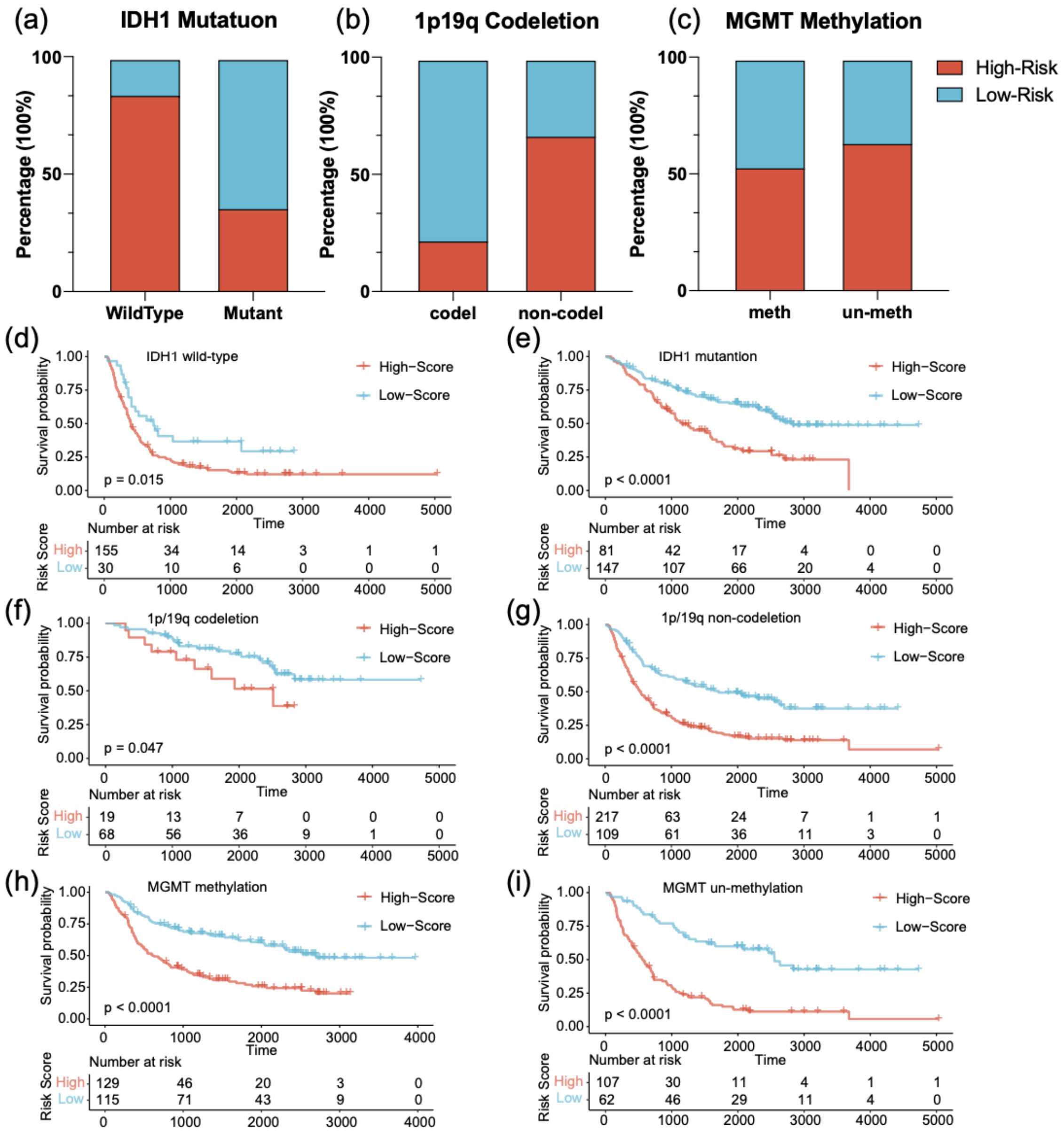

Figure S4: Validation the prognostic performance of patients carrying wild-type IDH1 genes, 1p19q codeletion or MGMT methylation. (a) IDH1 wild-type (WT) and mutation (Mutant) proportions in High Risk or Low Risk. (b) Chromosome 1p19q codeletion or not (Non) proportions in different risk groups. (c) MGMT methylation or not proportions in different risk groups. Kaplan-Meier curves of OS in IDH1 WT (d), IDH1 mutant (e), 1p19q codeletion (f), 1p19q non-codeletion (g), MGMT methylation (h) and MGMT un-methylation (i) based on risk score.
