# Supplementary material for: Upregulation of the APOBEC3 Family Is Associated with a Poor Prognosis and Influences Treatment Response to Raf Inhibitors in Low Grade Glioma
Source: Int J Mol Sci. 2021 Sep 27;22(19):10390. doi: 10.3390/ijms221910390 (PMC8508917; doi:10.3390/ijms221910390)
Supplement: Supplementary file 1 [file ijms-22-10390-s001.zip › Supplementary materials S5.pdf]

Supplementary Figures S5

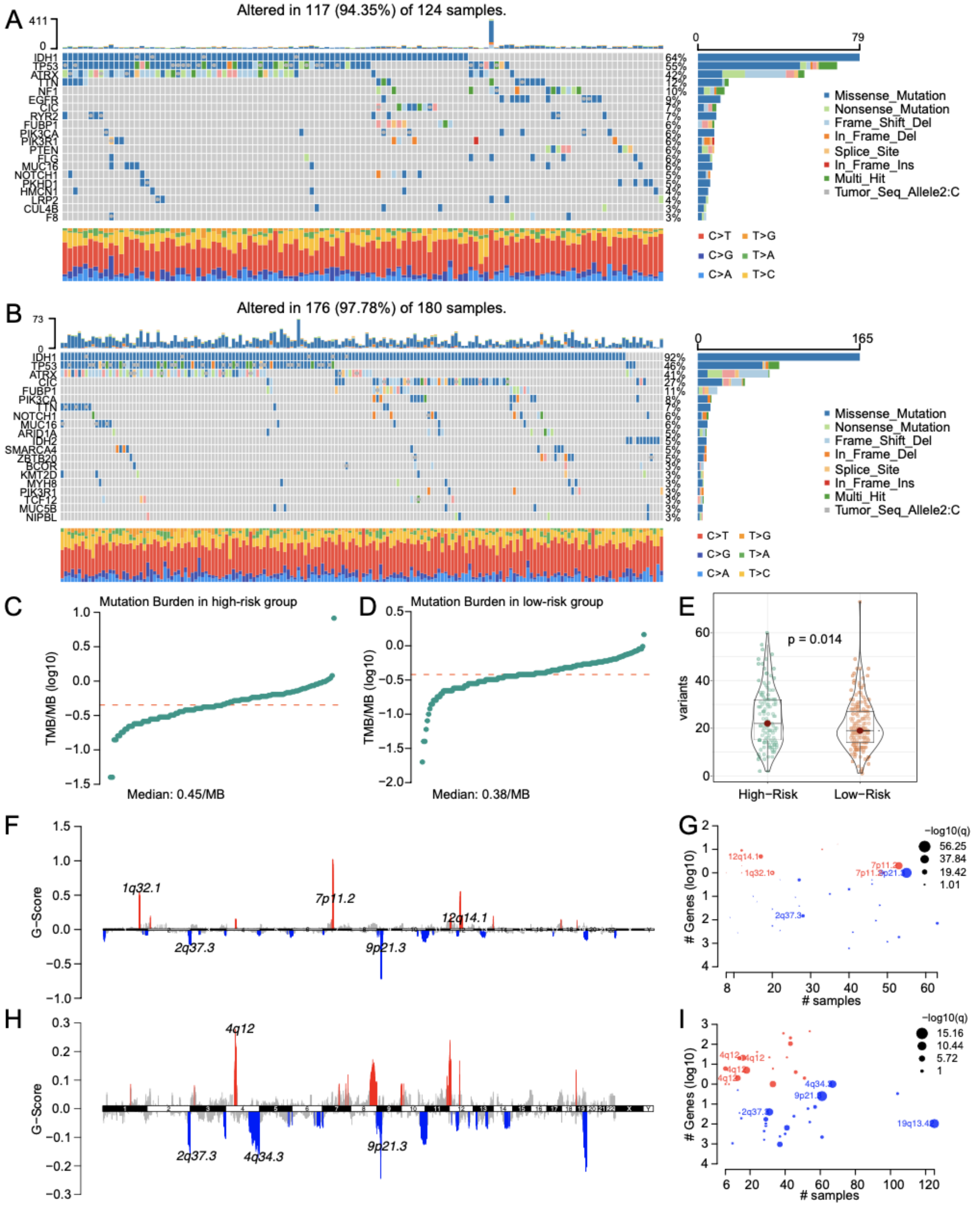

Figure S5. Molecular characteristics of different APOBEC3 subgroups. Top 20 genes with significant mutation in patients with (A) high and (B) low expression of APOBEC3 family and their proportion. The mutation burden in patients with (C) high and (D) low expression of APOBEC3 family. (E) The frequency of mutation events was different between high

and low expression group. (F, G) Copy number variation in LGG patients with high expression of APOBEC3 family. (H, I) Copy number variation in LGG patients with low expression of APOBEC3 family. The red region represents amplification and blue region represents deletion.
