# Supplementary material for: Upregulation of the APOBEC3 Family Is Associated with a Poor Prognosis and Influences Treatment Response to Raf Inhibitors in Low Grade Glioma
Source: Int J Mol Sci. 2021 Sep 27;22(19):10390. doi: 10.3390/ijms221910390 (PMC8508917; doi:10.3390/ijms221910390)
Supplement: Supplementary file 1 [file ijms-22-10390-s001.zip › Supplementary materials S6.pdf]

Supplementary Figures S6

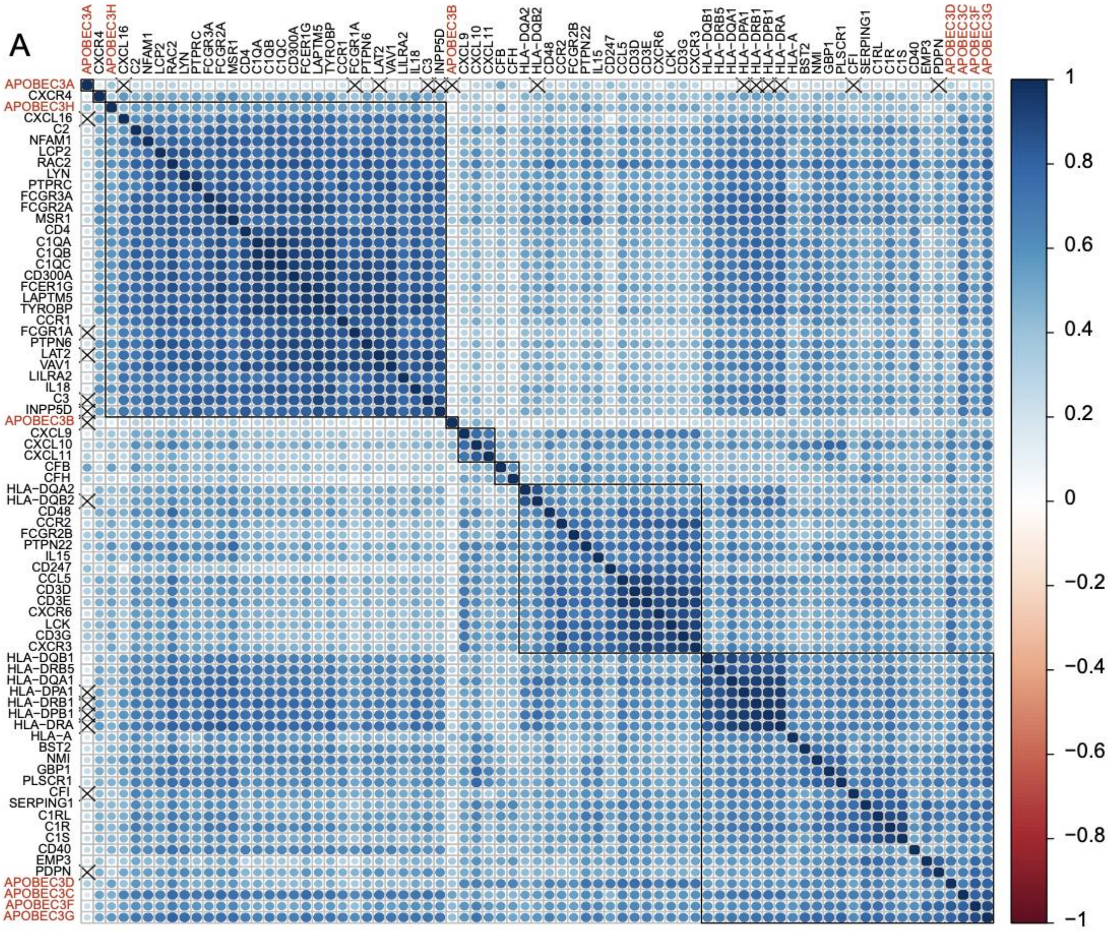

Figure S6: Correlation coefficients between APOBEC3 family and immune related genes annotated by MSigDB database. Cluster analysis is carried out according to the ‘hclust’ method of ‘corrplot’ package.
