# Supplementary material for: Upregulation of the APOBEC3 Family Is Associated with a Poor Prognosis and Influences Treatment Response to Raf Inhibitors in Low Grade Glioma
Source: Int J Mol Sci. 2021 Sep 27;22(19):10390. doi: 10.3390/ijms221910390 (PMC8508917; doi:10.3390/ijms221910390)
Supplement: Supplementary file 1 [file ijms-22-10390-s001.zip › Supplementary materials S7.pdf]

Supplementary Figures S7

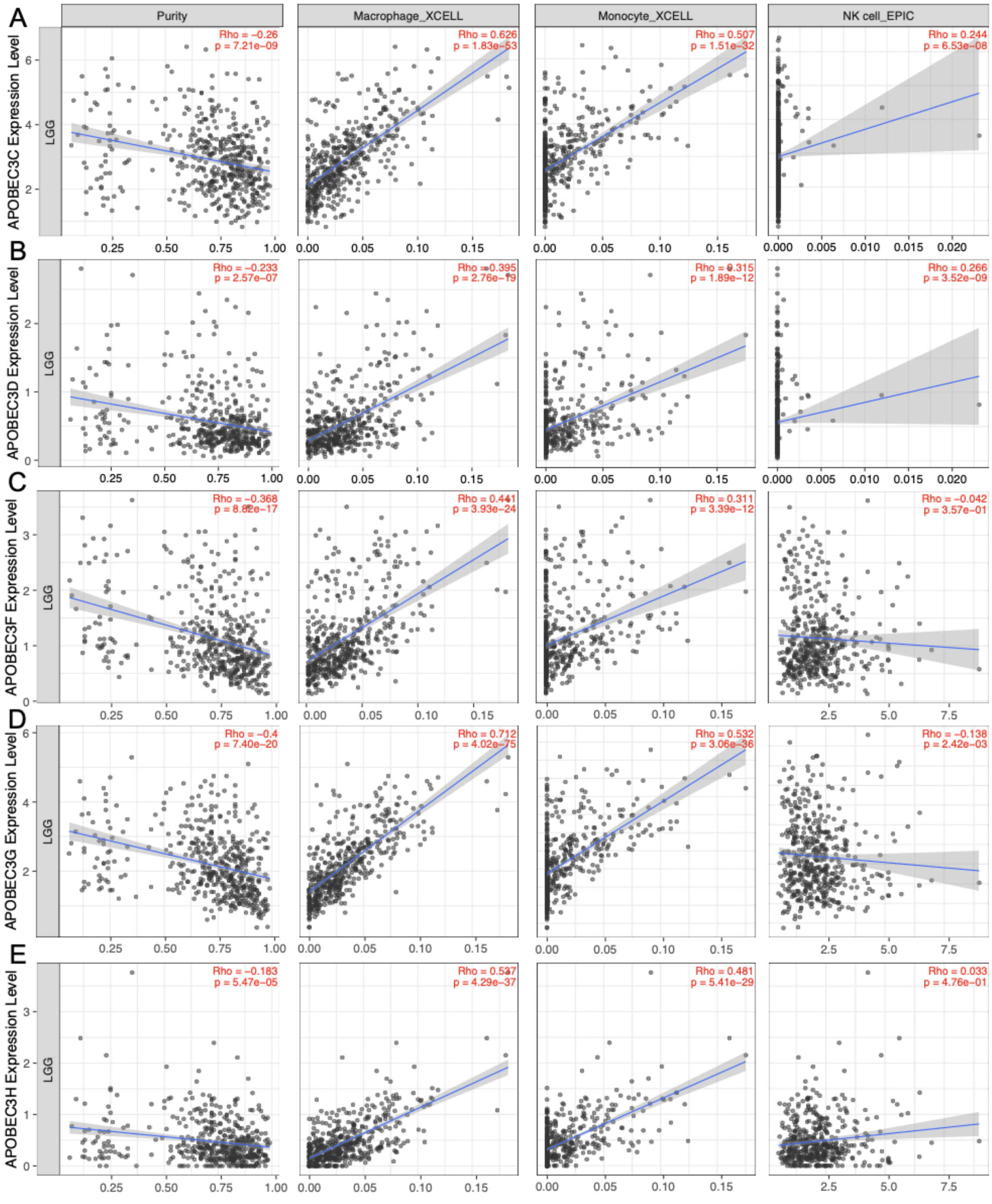

Figure S7: The expression of (A) APOBEC3C, (B)APOBEC3D, (C) APOBEC3F, (D) APOBEC3G and (E) APOBEC3H is associated with macrophage, monocytes and NK cells infiltration in low-grade glioma solid tumor.
