# Supplementary material for: Upregulation of the APOBEC3 Family Is Associated with a Poor Prognosis and Influences Treatment Response to Raf Inhibitors in Low Grade Glioma
Source: Int J Mol Sci. 2021 Sep 27;22(19):10390. doi: 10.3390/ijms221910390 (PMC8508917; doi:10.3390/ijms221910390)
Supplement: Supplementary file 1 [file ijms-22-10390-s001.zip › Supplementary materials S8.pdf]

Supplementary Figures S8

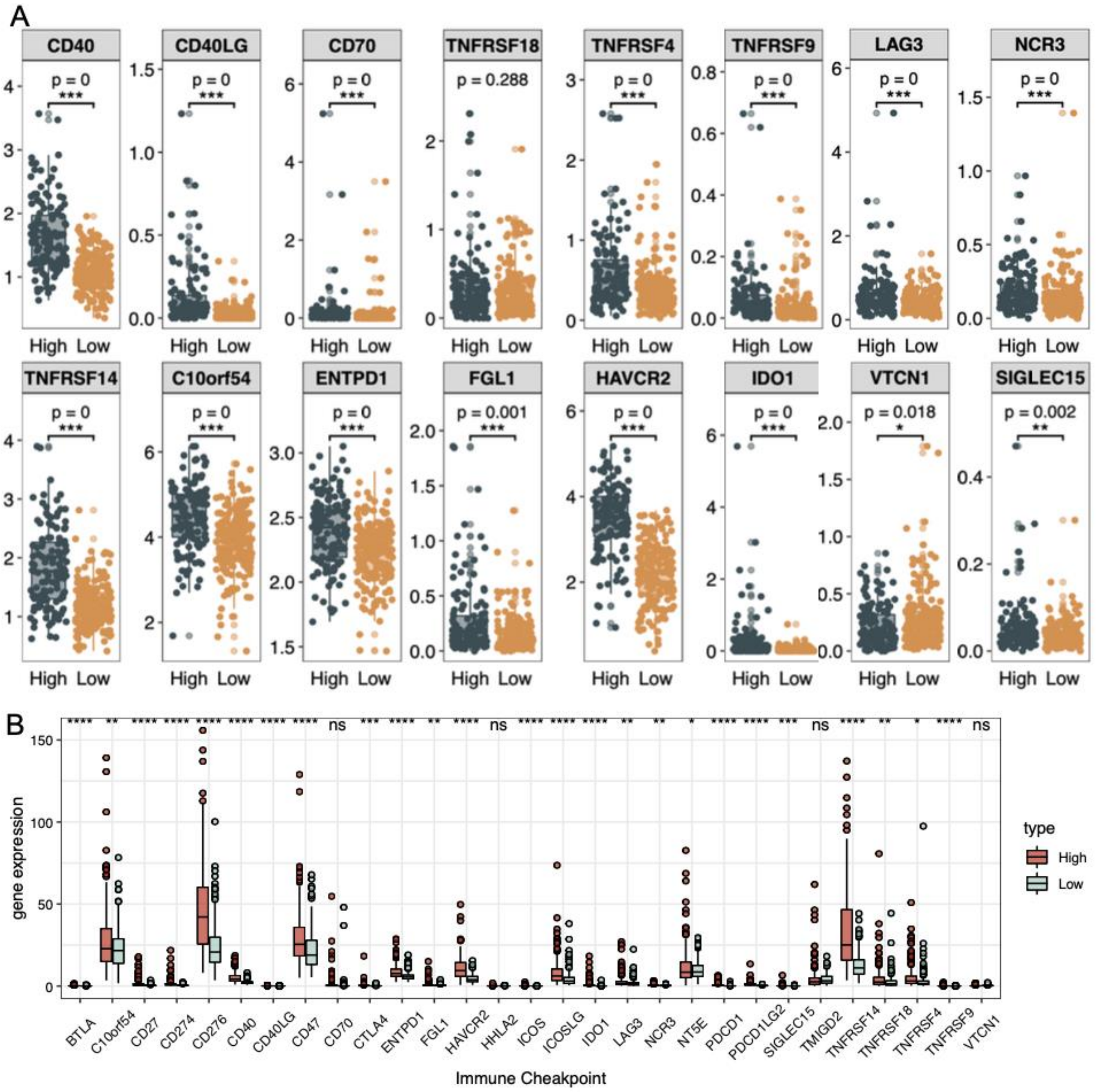

Figure S8: The relationship between APOBEC3 expression and immune checkpoints landscape in low grade glioma. (A) Expression of remaining immune checkpoints in TCGA dataset. (B) Expression of 29 immune checkpoints in CGGA dataset.
