# Supplementary material for: Upregulation of the APOBEC3 Family Is Associated with a Poor Prognosis and Influences Treatment Response to Raf Inhibitors in Low Grade Glioma
Source: Int J Mol Sci. 2021 Sep 27;22(19):10390. doi: 10.3390/ijms221910390 (PMC8508917; doi:10.3390/ijms221910390)
Supplement: Supplementary file 1 [file ijms-22-10390-s001.zip › Supplementary Table.pdf]

Supplementary Table

Table S1. Baseline data sheet for TCGA and CGGA data.

Characteristic	TCGA		CGGA
	Training Set	Internal validation	External validation
PRS_type , No. (%)			
Primary			248 (60%)
Recurrent			165 (40%)
Grade , No. (%)			
WHO II	157 (49%)	70 (50%)	96 (23%)
WHO III	163 (51%)	69 (50%)	161 (39%)
WHO IV			156 (38%)
Gender , No. (%)			
Female	146 (46%)	69 (50%)	182 (44%)
Male	174 (54%)	70 (50%)	231 (56%)
Age, mean ± SD	41 (33 to 52)	39 (31 to 52)	43.01 ± 12.36
Radio , No. (%)			
No	113 (35%)	61 (44%)	85 (21%)
Yes	207 (65%)	78 (56%)	328 (79%)
Chemo , No. (%)			
No			89 (22%)
Yes			324 (78%)
